# Whole-exome sequencing and genome-wide evolutionary analyses identify novel candidate genes associated with infrared perception in pit vipers

**DOI:** 10.1038/s41598-020-69843-w

**Published:** 2020-08-03

**Authors:** Na Tu, Dan Liang, Peng Zhang

**Affiliations:** State Key Laboratory of Biocontrol, College of Ecology and Evolution, School of Life Sciences, Higher Education Mega Center, Sun Yat-Sen University, Guangzhou, 510006 China

**Keywords:** Evolution, Molecular evolution

## Abstract

Pit vipers possess a unique thermal sensory system consisting of facial pits that allow them to detect minute temperature fluctuations within their environments. Biologists have long attempted to elucidate the genetic basis underlying the infrared perception of pit vipers. Early studies have shown that the *TRPA1* gene is the thermal sensor associated with infrared detection in pit vipers. However, whether genes other than *TRPA1* are also involved in the infrared perception of pit vipers remains unknown. Here, we sequenced the whole exomes of ten snake species and performed genome-wide evolutionary analyses to search for novel candidate genes that might be involved in the infrared perception of pit vipers. We applied both branch-length-comparison and selection-pressure-alteration analyses to identify genes that specifically underwent accelerated evolution in the ancestral lineage of pit vipers. A total of 47 genes were identified. These genes were significantly enriched in the ion transmembrane transporter, stabilization of membrane potential, and temperature gating activity functional categories. The expression levels of these candidate genes in relevant nerve tissues (trigeminal ganglion, dorsal root ganglion, midbrain, and cerebrum) were also investigated in this study. We further chose one of our candidate genes, the potassium channel gene *KCNK4*, as an example to discuss its possible role in the infrared perception of pit vipers. Our study provides the first genome-wide survey of infrared perception-related genes in pit vipers via comparative evolutionary analyses and reveals valuable candidate genes for future functional studies.

## Introduction

Infrared perception is a notable evolutionary adaptation of snakes that helps snakes respond to the external environment during their nocturnal activities and prey on warm-blooded animals^1–3^. Pit vipers are the best at sensing and utilizing infrared signals among all snakes^4–6^. They have the most sensitive infrared sensory system, which can detect minute temperature fluctuations as low as 0.001 °C in their immediate surroundings^7^. This peculiar infrared perception ability enables pit vipers to detect and locate warm-blooded animals with high precision in the dark, which makes them one of the most successful predators in nature^8–10^.

Biologists have long sought to investigate the biological basis of infrared perception in pit vipers. Previous anatomical and neurophysiological studies have accumulated substantial data explaining the detection and transduction mechanism of pit viper infrared perception^6,11–17^. We now know that the infrared perception of pit vipers is initiated by the heat stimulation of a thin sensory membrane embedded in the facial loreal pit, a highly specialized organ located between the eye and nostril^11,12^. The sensory membrane is innervated by afferent fibres of trigeminal nerve branches that transduce infrared signals to a particular nucleus in the hindbrain^13–15^. The efferents of the nucleus then project to the optic tectum, where infrared information is integrated with visual information to construct a thermal image (also known as “infrared vision”)^16,17^. This complex infrared sensory system must involve many genes at the genetic level. However, the genetic basis of the system that is involved in the infrared perception of pit vipers is far from defined and requires more attention. Recently, a study by Gracheva et al.^18^ showed that a particular gene, *TRPA1*, is highly expressed in the trigeminal ganglion (TG) of pit vipers. This gene encodes a calcium ion channel that can respond to temperature changes caused by infrared stimulation. Thus far, *TRPA1* is the only gene that has been shown to be involved in the infrared perception of pit viper snakes.

In pit vipers (and other infrared-sensitive snakes such as pythons and some boas^6^), the expression level of *TRPA1* in the TG is much higher than that in the dorsal root ganglion (DRG), which is not observed in infrared-insensitive snakes^18^. This difference in expression was the cornerstone of the transcriptome profiling analysis that led to the discovery of *TRPA1*. This strategy is able to reveal direct thermal sensor proteins that are involved in the infrared perception of snakes. However, it may not be able to identify genes involved in pit organ evolution, signal amplification, or information integration (if any) during infrared perception because such genes may not exhibit considerable changes in expression in different nerve tissues.

Taxonomically, pit vipers (Crotalinae) belong to the family Viperidae. There are three major clades of Viperidae: true vipers (Viperinae), pit vipers (Crotalinae), and Fea’s vipers (Azemiopinae), among which Viperinae diverged from the others first, followed by Azemiopinae and Crotalinae^19,20^. Notably, true vipers and Fea’s vipers do not have pit organs, and early animal behavior studies have found that these snakes are unable to sense infrared radiation^21,22^. These two viper groups are rather similar to pit vipers in many aspects, such as their morphology, venom, and lifestyle, but do not possess infrared perception ability^23^. Therefore, the infrared perception ability of pit vipers must have evolved after they diverged from the other two types of vipers.

Phenotype-associated genes often exhibit a particular evolutionary pattern in taxa with that phenotype compared to their sister taxa without the phenotype^24^. For example, the “language gene” *FOXP2* underwent accelerated evolution in the human lineage after its separation from the lineage leading to chimpanzees and bonobos, our closest relatives^25–27^; the echolocation gene *Prestin* shows convergent evolution in echolocating bats and whales^28–30^. Such comparative evolutionary analyses are an effective strategy for searching for phenotype-associated genes. Notably, our earlier work showed that the thermoreceptor gene *TRPA1* underwent strong accelerated evolution in pit vipers but not in true vipers^31^. In contrast, *TRPV1*, another TRP gene that is not associated with infrared perception, shows no difference in the rate of evolution between pit vipers and true vipers. Moreover, *TRPA1* contains many amino acid (AA) residues that are unique in pit vipers but evolutionarily conserved in other snakes^31^. We hypothesize that if there are other genes involved in the infrared perception of pit vipers, these genes may exhibit a similar evolutionary pattern to *TRPA1*. This particular evolutionary pattern could be used as a screening criterion to search for potential genes associated with infrared perception in pit vipers, which may compensate for the limitations of transcriptome profiling analysis.

A number of snake genomes have been sequenced and released in public databases^32–37^, including the genomes of members of both Viperinae and Crotalinae. However, representative genomes from Azemiopinae are lacking, hindering genome-wide comparative studies for the identification of infrared perception-related genes. In this study, we performed whole-exome capture sequencing to supplement the genomic data for the three subfamilies of Viperidae and other related snake species. Based on these new genomic data, we conducted a genome-wide survey to search for novel candidate genes related to the infrared perception of pit vipers. Our comparative evolutionary analyses identified 47 candidate genes with evolutionary patterns similar to that of *TRPA1*, which may be associated with infrared perception in pit vipers. Most of these genes are associated with ion transmembrane transporter activity. We hope that the genome-wide identification and evolutionary characterization of novel candidate genes will contribute to the further elucidation of the genetic basis of the infrared perception of pit vipers.

## Results and discussion

### Coding sequence (CDS) data

In this study, we sequenced the whole coding sequences of a total of 10 snake species (see Supplementary Table S1 for sample details) by sequence capture. The sequence capture experiment was performed by using a custom-designed capture probe set (Agilent Custom SureSelect Kit) to selectively target the coding regions of ~ 20,000 snake protein-coding genes. High-throughput sequencing was performed on an Illumina HiSeq X-10 sequencer in 150-bp paired-end mode, and the numbers of read pairs among the samples ranged from 27 to 56 million (40.25 million on average). Approximately 23% of the reads could be mapped to the 21,331 reference CDSs of *Deinagkistrodon acutus*^32^*,* and the on-target percentage across the ten snake samples varied from 9.8 to 31.3%. Based on these data, we were able to reconstruct over 20,000 CDSs across all the samples (average = 21,197) with an average CDS data completeness of 92.24% (Supplementary Table S1). We compared the obtained CDSs of our *Ophiophagus hannah* sample with the sequences of the previously published king cobra genome^38^ and found 99% sequence similarity, suggesting that the sequence quality of our CDS data is high. By combining these newly generated CDS data with the 9 published snake genomes (Supplementary Table S2), we produced a dataset consisting of 21,331 orthologous gene groups (OGGs) for the 19 snake species, including eight pit vipers, one Fea’s viper, two true vipers, and representative species from five other snake families.

### General pattern of gene evolution in viper snakes

**Figure 1:**
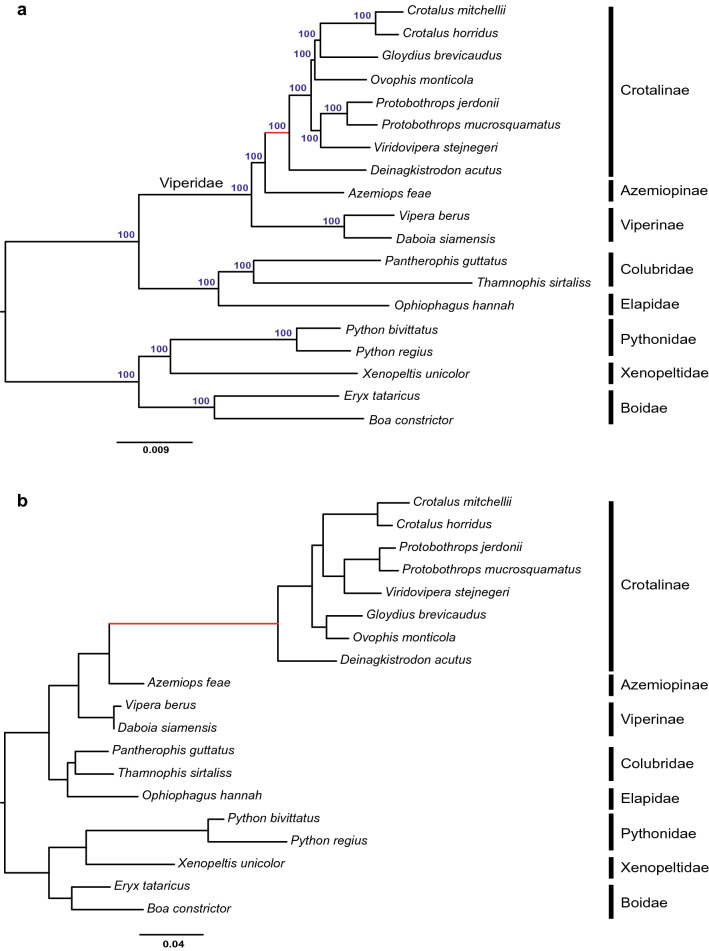
The genome phylogenetic tree (**a**) and the *TRPA1* gene tree (**b**). Numbers on the branches are ML bootstrap support values. The ancestral branch of pit vipers are marked in red.

To better understand the evolutionary history of the viper snakes included in this study and provide an accurate phylogenetic framework for comparative analysis, we first constructed a genome-scale phylogenetic tree for the 19 snake species based on the concatenated protein sequences of all 21,331 OGGs. The resulting tree (Fig. 1a) was highly resolved, with all nodes exhibiting 100% bootstrap support, and was consistent with a recent snake phylogeny^19,20^. In this tree, Viperidae is monophyletic; Azemiopinae (Fea’s vipers) is the sister group of Crotalinae (pit vipers), and Viperinae (true vipers) is the sister group of both of the first two groups. Notably, within Viperidae, the branch lengths from the terminal tips to the root node of Viperidae are rather uniform across the three subfamilies, suggesting that most of the protein-coding genes evolved at a similar pace among the three types of viper snakes. In contrast, in the gene tree of the infrared perception-related *TRPA1* gene (Fig. 1b), the branch lengths of Crotalinae are considerably longer than those of the other two viper subfamilies.

### Identification of candidate genes associated with infrared perception in pit vipers

**Figure 2:**
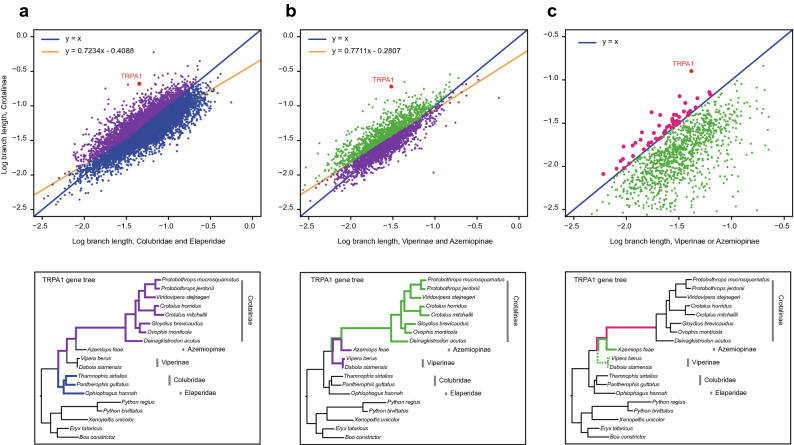
Genome-wide survey of candidate genes for infrared perception in pit vipers based on branch length analysis. The analysis includes three comparisons performed to search for genes that exhibit similar evolutionary patterns to *TRPA1*. The *TRPA1* gene tree is provided as an example below every search step. (**a**) The average root-to-tip branch length of pit vipers (purple branches) compared to the average root-to-tip branch length of the Colubridae + Elapidae clade (blue branches). Each dot represents one gene. Purple dots are retained for the next step of filtering. (**b**) The average root-to-tip branch length of pit vipers (green branches) compared to that of other vipers (purple branches). Green dots are retained for the next step of filtering. (**c**) The ancestral branch length of pit vipers (magenta branches) compared to the longest root-to-tip branch length of other viper snakes (green branches). The longest branch is highlighted by the solid green line, whereas the dashed green lines represent shorter branches. Magenta dots represent the final retained genes.

In the *TRPA1* gene tree, the ancestral branch of pit vipers is considerably longer (more than three times) than the sister branch connecting to true vipers, indicating that pit vipers have evolved considerably faster than all other snakes (exemplar tree; Fig. 2). We thus first utilized this special branch length pattern to search for potential infrared perception-related genes among the 21,331 OGGs.

In this analysis, individual gene trees were reconstructed for all 21,331 OGGs by maximum likelihood inference using the obtained genome tree as a topology constraint. We used a three-step filtering strategy to gradually narrow our candidate gene pool from these gene trees. First, we wanted to screen out genes that evolved more rapidly in Crotalinae (pit vipers) than in Colubridae and Elapidae (outgroup snakes). To do so, we considered the average branch length from the common ancestral node to all termini of a given snake group as the average evolution rate of the group. For example, starting from the common ancestral node of Crotalinae + Colubridae + Elapidae, the average branch length to all Crotalinae termini (Fig. 2a; purple branches in the phylogram) and the average branch length to all Colubridae + Elapidae termini (Fig. 2a; blue branches in the phylogram) were regarded as the average evolution rates of Crotalinae and the outgroup, respectively. For both snake groups, the average branch lengths were calculated and plotted. The genes located above the regression line and the 1:1 line (evolving faster in pit vipers than in outgroup snakes) were retained (Fig. 2a). Second, the retained genes were further filtered by comparing the average branch length of Crotalinae with that of Viperinae and Azemiopinae (infrared-insensitive viper snakes) (Fig. 2b). Genes located below the 1:1 geometric and regression lines were filtered out. By doing so, we aimed to ensure that the selected candidate genes have evolved faster in pit vipers than in the other two types of viper snakes. Third, we plotted the ancestral branch length of pit vipers and the longest branch length leading to the Viperinae or Azemiopinae terminal and retained genes located above the 1:1 line (Fig. 2c). This step was performed to identify genes that experienced accelerated evolution on the ancestral branch of pit vipers. The three-step filtering process led to the identification of a total of 51 candidate genes. Finally, we reconstructed ML trees for these 51 genes without topology constraints and repeated the above three filtering steps. Among the 51 candidate genes, 38 genes passed the filtration procedure (Supplementary Table S3).

We manually inspected the gene tree of these 38 candidate genes to check for pit-viper-specific signatures of accelerated evolution (Supplementary Fig. S1). All these gene trees exhibit similar evolutionary patterns to *TRPA1,* although the patterns are somewhat less conspicuous than that of *TRPA1*. For example, the *KCNK4* gene displays a relatively longer length along the common ancestral branch of pit vipers, and the total evolutionary branch length is significantly longer than those of all other snake lineages; the *KCNH8* gene displays a longer branch length not only in pit vipers but also in pythons (another group of snakes with infrared perception ability), which may imply a close association with their common characteristic of infrared perception ability; a hypothetical gene with the ID XB-GENE-5885309 has evolved much faster in pit vipers than in adjacent vipers. These shared evolutionary patterns similar to *TRPA1* suggest that other genes might also participate in the specialized trait of pit vipers, the infrared perception.

**Figure 3:**
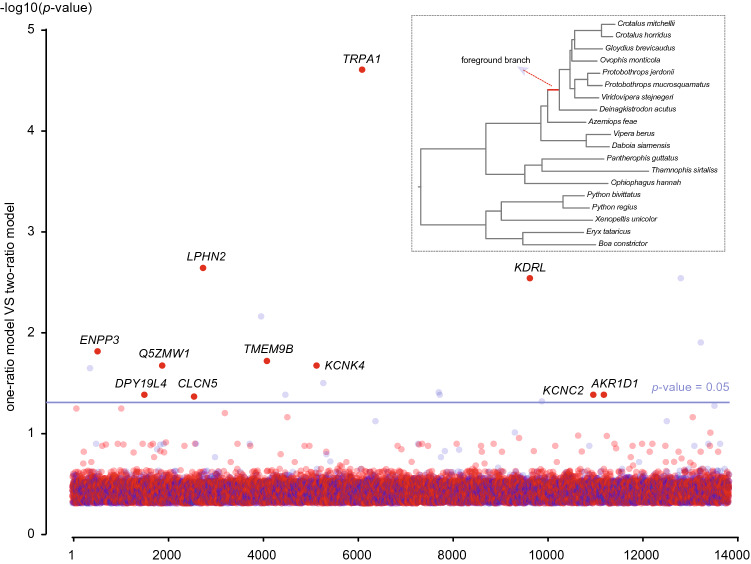
Genome-wide survey of candidate genes for infrared perception in pit vipers based on selection pressure analysis. The one-ratio model is compared with the two-ratio model, and their relative fit is evaluated using likelihood ratio tests with statistical correction. The ancestral branch of the pit viper lineage is set as the foreground branch. Genes with ω1 > ω0 are indicated in red, whereas genes with ω1 < ω0 are indicated in light blue. Each dot represents an individual gene, and the x-axis represents the numerical order of the genes.

In addition to the branch length analysis, we performed a genome-wide scan based on selection pressure comparison. As an infrared perception-related gene of pit vipers, *TRPA1* experienced significantly different selection pressure, showing an elevated dN/dS value (the ratio of nonsynonymous to synonymous substitutions) in the ancestral branch of pit vipers^31^. Thus, we compared the one-ratio model and the two-ratio model for all 21,331 OGGs by PAML^39^. The one-ratio model assumes that all branches in the phylogeny have the same ω value (dN/dS), while the two-ratio model assumes two different ω values within the phylogeny: one for the ancestral branch of pit vipers (ω_1_) and one for the remaining branches of the tree (ω_0_). If the two-ratio model is significantly better than the one-ratio model for a gene, the gene experienced significantly different selection pressure in the ancestral branch of pit vipers. The selection pressure analysis identified a total of 11 genes with significantly elevated ω values in the ancestral branch of pit vipers compared to the background branches (ω_1_ > ω_0,_ with FDR-adjusted P-value < 0.05; Fig. 3 and Supplementary Tables S4 and S5).

Combining the 11 candidate genes from the selection analysis and the 38 candidate genes from the branch length analysis, we identified a total of 47 candidate genes that may be related to the infrared perception of pit vipers. These genes are *TRPA1*, *KCNK4*, *WAPAL*, *NOC4L*, *Q5ZLE7*, *ENPP3*, *KCNH8*, *LPHN2*, *TMEM9B*, *XB-GENE-5885309*, *CLCN5*, *DPY19L4*, *GK5*, *KDRL*, *ADAMTS2*, *AKR1D1*, *HRH2*, *C1QL2*, *CAST*, *HABP4*, *LHX3*, *SLC6A16*, *ZNF703*, *ZNT6*, *CERS6*, *COQ5*, *F1NPK8*, *FAM58A*, *KCNC2*, *KCNK17*, *METTL20*, *Q5ZMW1*, *SETD7*, *SLC45A1*, *VWA7*, *WWOX*, *AGTR1*, *CHMP6*, *CRYAB*, *CYBB*, *DECR2*, *LRRC3*, *MYOT*, *NDUFB8*, *SKA1*, *SLC5A1*, and *ZGC_136493*. Among these genes, only two (*TRPA1* and *KCNK4*) were identified simultaneously by both filtered schemes. Detailed information on all candidate genes is given in Supplementary Table S6.

### Characteristics of the candidate genes

To further explore the possible associations between the identified genes and the infrared perception function in pit vipers, we retrieved Gene Ontology (GO) terms for the 47 candidate genes under three major categories: biological process (BP), cellular component (CC), and molecular function (MF). Additionally, we performed GO enrichment analysis to test whether our candidate genes were significantly enriched in specific GO terms. The GO enrichment analysis showed that five BP GO terms, two CC GO terms, and four MF GO terms were significantly enriched (Table 1). A GO term of the CC category (integral component of membrane) included the most genes (17 of the candidate genes). Remarkably, all enriched GO terms of the MF category were related to ion channel genes, including the *TRPA1*, *KCNK4*, *KCNH8*, *KCNK17*, *KCNC2*, *CYBB*, and *CRYAB* genes. Ion channel proteins are crucial in the thermosensation of vertebrates^40^. Because our screening scheme targeted infrared perception, these ion channel genes are unlikely to have been randomly identified. It is worth mentioning that we found that the two genes (*TRPA1* and *KCNK4*) that were identified by both the selection pressure and branch length analyses were associated with the GO term ‘temperature-gated cation channel activity (GO: 0097604)’. This GO term was significantly enriched (468.9-fold), with the lowest corrected *P*-value of 4.4e−6.

**Table 1:** Over-represented GO categories among the 47 candidate genes.

Categories	Description	Gene number	Corrected *P* value	Enrichment fold	Genes
**Biological progress**					
GO:0071805	Potassium ion transmembrane transport	4	1.10E−04	15.9	KCNK17, KCNH8, KCNK4, KCNC2
GO:0034765	Regulation of ion transmembrane transport	4	8.10E−05	17.3	KCNK17, CYBB, KCNH8, KCNK4
GO:0030322	Stabilization of membrane potential	2	5.00E−04	60	KCNK17, KCNK4
GO:0042542	Response to hydrogen peroxide	2	5.00E−03	18.8	CRYAB, TRPA1
GO:0014070	Response to organic cyclic compound	2	4.70E−03	19.6	KCNC2, TRPA1
**Cellular component**					
GO:0005887	Integral component of plasma membrane	12	1.40E−05	4.2	AGTR1, CLCN5, CYBB, ENPP3, HRH2, KCNK17, KCNK4, KCNC2, KCNH8, SLC5A1, SLC6A16, TRPA1
GO:0016021	Integral component of membrane	17	1.70E−02	1.6	NDUFB8, TMEM9B, AGTR1, CERS6, CLCN5, CYBB, DPY19L4, ENPP3, LRRC3, NOC4L, KCNK17, KCNK4, KCNC2, SLC45A1, SLC5A1, SLC6A16, TRPA1
**Molecular function**					
GO:0005244	Voltage-gated ion channel activity	3	4.20E−05	44	KCNK17, CYBB, KCNK4
GO:0097604	Temperature-gated cation channel activity	2	4.40E−06	468.9	TRPA1, KCNK4
GO:0022841	Potassium ion leak channel activity	2	5.20E−04	58.6	KCNK17, KCNK4
GO:0005267	Potassium channel activity	2	2.80E−03	25.3	KCNK17, KCNK4

**Figure 4:**
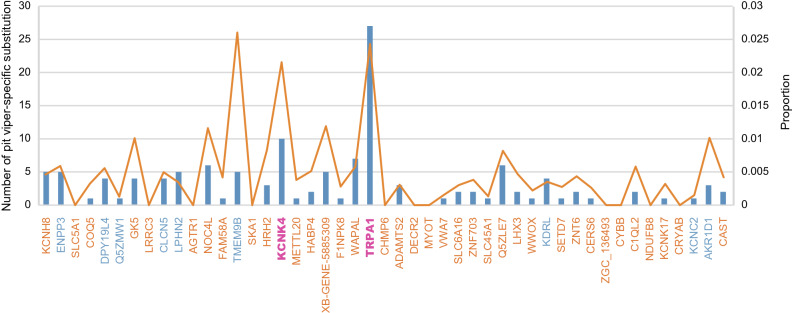
The number and proportion of pit viper-specific amino acid substitutions for the 47 candidate genes. The blue bars are numbers, and the orange lines are proportions. The genes identified by branch length analysis are orange, the genes identified by selection analysis are blue, and the two genes (*TRPA1* and *KCNK4*) identified by both analyses are magenta.

Residue substitutions at evolutionarily conserved positions are often signs of the functional alteration of proteins. Therefore, we calculated the number of pit viper-specific amino acid (AA) substitutions for each candidate gene and used a branch-specific site model to test whether these substitution sites are under positive selection. A pit viper-specific substitution is defined as an amino acid site that is in one state for all other snake groups but is substituted to another state in pit vipers. Thirty-six of the 47 candidate genes possess at least one pit viper-specific substitution; specifically, the *TRPA1*, *KCNK4*, *WAPAL*, *NOC4L*, *Q5ZLE7*, *ENPP3*, *KCNH8*, *TMEM9B*, *XB-GENE-5885309*, *CLCN5*, *DPY19*, *GK5*, *KDRL*, and *ADAMTS2* genes present more than five pit viper-specific AA substitutions (Fig. 4 and Supplementary Table S6). Most of these substitution sites are also under positive selection in the ancestral branch of pit vipers (Supplementary Table S6). The *TRPA1* gene possesses the highest number of pit viper-specific AA substitutions (27 in total) in its protein sequence. Among the remaining candidate genes, the *KCNK4* gene possesses the highest number of pit viper-specific AA substitutions (10 in total), and the proportion is comparable to that for *TRPA1* (Fig. 4). When considered according to the proportion of pit viper-specific AA substitutions, *TMEM9B* is the most prominent gene among all candidate genes. This gene is related to an obsolete signal transducer activity (GeneCards, https://www.genecards.org) and exhibits a functional interaction with *CLCN5*, a gene that is also present in our candidate gene set. In summary, the functional properties of many of our candidate genes suggest that they may play a role in the infrared perception of pit vipers, but their functional mechanisms need to be explored in more detail.

### Expression levels of candidate genes in cerebrum, midbrain, TG and DRG nerve tissues

**Figure 5:**
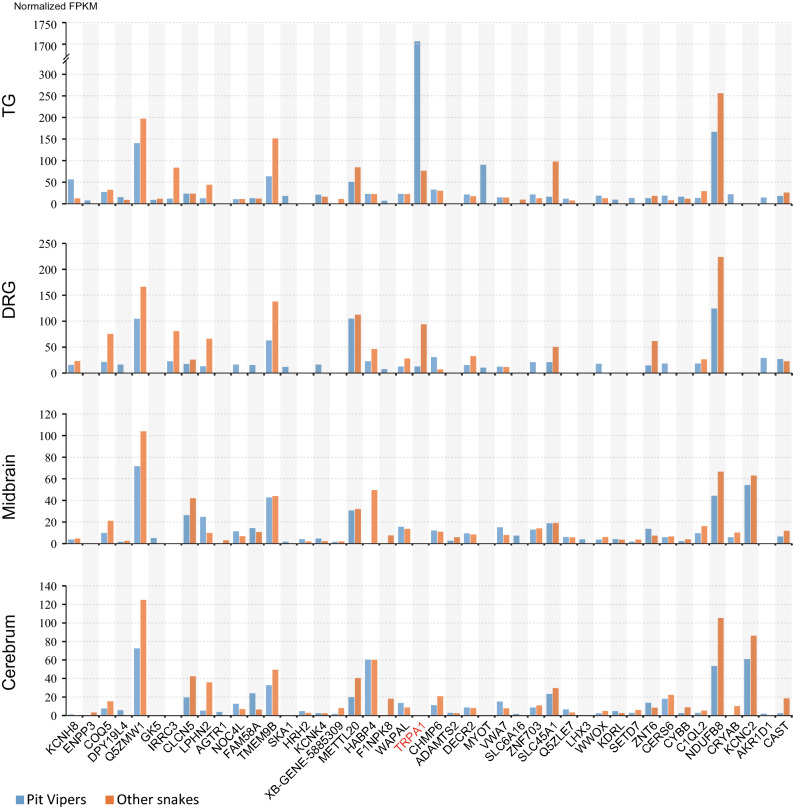
Expression levels of candidate genes in four nerve tissues of pit vipers and other infrared-insensitive snakes: trigeminal ganglion (TG), dorsal root ganglion (DRG), cerebrum, and midbrain.

Our comparative evolutionary analyses identified a total of 47 candidate genes. The investigation of the expression levels of these candidate genes in relevant nerve tissues is necessary to elucidate their functional roles in infrared perception in pit vipers. Therefore, we performed transcriptome profiling to investigate the expression levels of the candidate genes in the cerebrum, midbrain, TG, and DRG nerve tissues of both pit vipers and infrared-insensitive snakes (Fig. 5). In pit vipers, the TG contains sensory neurons that directly connect to the sensory membrane embedded in the facial pit. The DRG, midbrain, and cerebrum were used as comparative references to the TG. Expression levels were measured by the trimmed mean of M-values (TMM) normalized fragments per kilobase of transcript per million mapped reads (FPKM) values to compare the expression levels of different genes across different samples.

Among the 47 candidate genes, three genes (*KCNK17*, *SLC5A1*, and *ZGC_136493*) were not expressed in any of the four nerve tissues that we investigated. Among the remaining 44 genes, all but four genes (*AGTR1*, *ADAMTS2*, *LHX3*, and *KCNC2*) were expressed in the TG of pit vipers. When pit vipers were compared with infrared-insensitive snakes, nine genes were found to exhibit upregulated expression in the pit viper TG. They are *TRPA1*, *KCNH8*, *MYOT*, *KCNK4, ZNF703*, *KDRL*, *SETD7*, *CYBB*, and *CRYAB* (Fig. 5). For example, the expression level of *TRPA1* in the pit viper TG (FPKM 1707.7) is considerably higher than in the other three nerve tissues (DRG: 12.7; midbrain: 0; cerebrum: 0). The expression levels of *KCNH8* and *MYOT* in the pit viper TG and the other three nerve tissues are TG: 56.5; DRG: 15.6; midbrain: 3.8; cerebrum: 1.1 and TG: 90.5; DRG: 10.1; midbrain: 0; cerebrum: 0, respectively. The expression level of *KCNK4* in the TG is also higher than the levels in the other three nerve tissues, but the expression differences are small (TG: 21.2; DRG: 16.3; midbrain: 4.7; cerebrum: 2.7). Detailed quantitative results are provided in Supplementary Table S7.

Among the eight upregulated genes (excluding *TRPA1*), the molecular functions of five genes (*ZNF703*, *KDRL*, *SETD7*, *CYBB*, and *CRYAB*) show no direct association with infrared perception based on the current knowledge of human orthologous proteins. Among the remaining three genes, *KCNH8* is a voltage-gated potassium channel gene involved in regulating neurotransmitter release and neuronal excitability^41^; *MYOT* encodes a cytoskeletal protein that plays an essential role in stabilizing thin filaments during muscle contraction^42^, and *KCNK4* functions as an outwardly rectifying channel and may be involved in regulating the input threshold in sensory neurons^43^. All of these functions are associated with infrared perception to some extent. The relatively higher expression of these genes in the trigeminal ganglion of pit vipers implied that they might function in the infrared perception of pit vipers.

### Possible functional mechanism with *KCNK4* as an example

Through genome-wide evolutionary analyses, we identified a number of genes that may function during the infrared perception of pit vipers. However, whether these genes are truly functional is still unclear. We thus attempted to obtain some clues regarding the function of these genes via protein structure simulation and functional inference. *KCNK4* was a repeatedly identified candidate gene in all of our evolutionary analyses. This gene has experienced accelerated evolution in the ancestral branch of pit vipers and accumulated large numbers of pit viper-specific amino acid mutations (Supplementary Fig. S2), and it exhibits upregulated expression in pit viper TG. Although the upregulated expression pattern of this gene in the pit viper TG was not significant (Fig. 5), the unique amino acid mutations distributed in critical regions of the protein may confer a functional advantage in the infrared perception of pit vipers. We, therefore, chose *KCNK4* as an example to perform protein structure simulation to further explore its possible functional mechanism.

**Figure 6:**
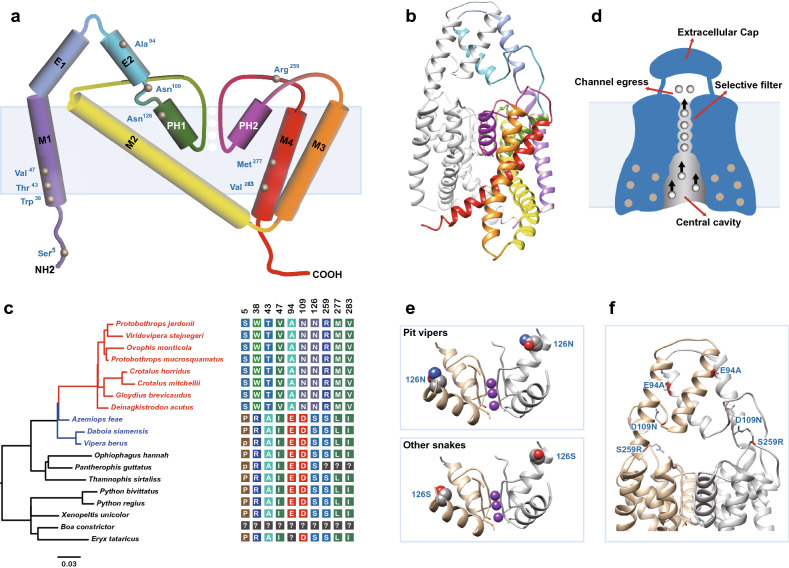
Protein structure simulation of the mutated pit viper KCNK4 channel. (**a**) Topology diagram of KCNK4 and the locations of the ten pit viper-specific amino acid substitutions. (**b**) Three-dimensional structure of the pit viper KCNK4 dimer predicted by homology modeling based on the human KCNK4 crystal structure. (**c**) The *KCNK4* gene tree of 19 snake species (pit vipers are red, and other vipers are blue) and a protein sequence alignment showing the ten pit viper-specific mutations (the full alignment is given in Supplementary Fig. S2). (**d**) Functional structure diagram of the KCNK4 channel. Five amino acid mutations (R38W, A43T, I47V, L277M, I283V; labeled with filled grey circles) are present in the central cavity. (**e**) Structure of the selective filter in the two conformational states of the proteins from pit vipers (126 N) and other snakes (126S). (**f**) Three amino acid mutations (E94A, D109N, S259R) distributed around the KCNK4 channel egress.

The protein structure simulation showed that the complete *KCNK4* channel is a homodimer in which each subunit comprises an extracellular cap that controls channel egress, four transmembrane helices (M1–M4) surrounding the central cavity, and two pore-helices (PH1 and PH2) forming the selectivity filter (Fig. 6a, b, d)^44^. Among the ten pit viper-specific amino acid mutations (Fig. 6c), three (R38W, A43T, I47V) are located in M1, and two (L277M, I283V) are located in M4. Earlier studies reported that amino acid mutations in the transmembrane helices of K_2P_ channels would affect channel gating, which may blunt the channel responses to various types of gating commands^45,46^. Therefore, we hypothesized that the five amino acid mutations in the transmembrane helices of pit viper KCNK4 might have a similar effect. Pore helices are vital components that play an essential role in maintaining the pore conformation of the *KCNK4* channel^44,47^. Mutations in the pore helices of various K_2P_ channels, such as *KCNK1*, *KCNK2*, *KCNK4,* and *KCNK9*, have been reported to dramatically affect the gating of the channels^45,48,49^. Pit viper *KCNK4* harbors one mutation (S126N) located in pore helix 1 (PH1). Protein structure simulation showed that the side chain of this amino acid residue points towards the central pore. In terms of space, the asparagine (N) side chain is bulkier than the serine (S) side chain, causing increased steric hindrance (Fig. 6e), which is also likely to have an impact on channel gating. Three additional amino acid mutations (E94A, D109N, S259R) are located in the extracellular cap helix, the upstream linker of PH1, and the downstream linker of PH2. Protein structure simulation showed that these three residues are distributed around the channel egress (Fig. 6f). Interestingly, in pit viper *KCNK4*, two negatively charged residues, Glu and Asp, are replaced with two noncharged residues, Ala and Asn (E94A, D109N), and the noncharged residue Ser is replaced by the positively charged residue Arg (S259R). Such replacements alter the charge environment of the channel egress from a negative state, which attracts potassium ions, to a positive state, which repels potassium ions, potentially also affecting the channel gating.

Early functional works showed that the *KCNK4* channel is involved in maintaining the resting membrane potential and is closely associated with neuron excitability^50–52^. This gene is usually coexpressed with heat-sensitive and cold-sensitive excitatory channels, such as *TRPV1*, *TRPM8*, and *TRPA1*, and functions as a silencer of these excitatory channels in sensory neurons^53–56^. It has been reported that downregulated expression or knockout of *KCNK4* in mice can increase neuronal sensitivity towards a given thermal stimulus, causing heat hyperalgesia or cold hypersensitivity in mice^53,54^. This is because the deletion of neuronal K^+^ channels in nociceptors will slow the depolarization of sensory neurons and prolong action potentials, thus amplifying painful sensations^53^. Our protein structure simulation analysis suggested that the mutations in pit viper *KCNK4* channel are likely to affect channel gating, which might blunt the channel responses to certain types of gating commands or alter the thermal or mechanical sensitivity of the channel. If these mutations make the thermal activation threshold of pit viper *KCNK4* increase, this channel will be less active at lower temperatures at which *TRPA1* is activated. This functional effect is similar to knockdown or knockout *KCNK4* in pit vipers’ infrared sensory neurons. If our functional speculation is correct, the pit vipers seem to use a mechanism similar to that underlies heat hyperalgesia in a mouse model generated by the deletion of *KCNK4* to increase their thermosensitivity. Of course, this hypothesized mechanism needs further confirmation with functional studies.

## Conclusions

Compared with other specialized sensory systems such as mammalian echolocation and primate trichromatic vision, the molecular underpinning of snake infrared perception has received relatively little attention. Before this study, researchers had only performed transcriptome differential expression analyses to identify infrared perception-related genes in pit vipers and obtained only one target gene, *TRPA1*. On the basis of whole-exome capture sequencing, we conducted the first genome-wide mining of candidate genes associated with infrared perception function in pit vipers through comparative evolutionary analyses. Our study identified a total of 47 genes that underwent accelerated evolution, specifically in the pit viper lineage, which exhibited extensive accumulated pit viper-specific amino acid changes in their protein sequences. Most of the candidate genes were enriched in GO terms functionally related to ion transmembrane transport, which is closely related to infrared perception. We also examined the expression levels of these candidate genes in four different types of nerve tissues of both pit vipers and infrared-insensitive snakes and found that eight genes exhibited upregulated expression in the pit viper trigeminal ganglion. Furthermore, we simulated the protein structure of one of our candidate genes, the potassium channel gene *KCNK4*, and inferred its possible functional mechanisms. This work represents the first attempt to systematically identify genes associated with pit viper infrared perception based on comparative genomic analysis. We hope that the gene sets reported in this study provide a valuable stepping stone for further functional research and shed new light on the genetic basis of infrared perception in pit vipers.

## Methods

### Taxon sampling

In this study, a total of 19 snake species were sampled for comparative genomic analysis, covering all 3 subfamilies of Viperidae and representatives from five other relevant snake families (Supplementary Table S2). Of these snake species, nine have genomic data available in public databases, including four infrared-sensitive crotalines (*Crotalus mitchellii*, *Crotalus horridus*, *Protobothrops mucrosquamatus*, and *Deinagkistrodon acutus*), one infrared-insensitive viper (*Vipera berus*), two infrared-insensitive colubrids (*Pantherophis guttatus* and *Thamnophis sirtalis*), one infrared-sensitive python (*Python bivittatus*) and one infrared-sensitive boa (*Boa constrictor*). In addition, we sampled another 10 snake species to conduct whole-coding-region sequence capture. These species included four Crotalinae species (*Gloydius brevicaudus*, *Protobothrops jerdonii*, *Viridovipera stejnegeri*, and *Ovophis monticola*) and one Azemiopinae species (*Azemiops feae*), one Viperinae species (*Daboia siamensis*), one Elapidae species (*Ophiophagus hannah*), one Pythonidae species (*Python regius*), one Xenopeltidae species (*Xenopeltis unicolor*) and one Boidae species (*Eryx tataricus*). This additional sampling supplements the missing Azemiopinae and enriches the sampling density of other families, especially pit vipers (Crotalinae).

All animal experiments in this study (including the RNA-seq experiments mentioned below) were performed in strict accordance with the guidelines and regulations developed by the China Council on Animal Care and Use. Animal processing procedures were approved by the Institutional Animal Care and Use Committee of Sun Yat-Sen University (permit number: 2018-023).

### Whole-exome capture and sequencing

Genomic DNA of the 10 snake species were extracted from muscle tissues using the DNeasy Blood & Tissue Extraction Kit (Qiagen). The extracted gDNA samples were randomly fragmented by NEBNext dsDNA fragmentase (New England Biolabs) to 200–400 bp. A total of 500 ng of fragmented gDNA was used for DNA library construction with the NEBNext Illumina DNA Library Prep Kit (New England Biolabs) according to the manufacturer’s protocol. We used the Custom Agilent SureSelectXT Target Enrichment System (Agilent Technologies) to capture whole coding regions from these libraries. The custom probes were designed at twofold tiling to target 21,331 snake CDSs for a total size of 32.1 Mb, based on the predicted exome of *Deinagkistrodon acutus*^32^. The hybridization enrichment experiments were performed using the SureSelectXT Reagent Kit (G9611A) according to the manufacturer’s protocol. After enrichment, hybridized libraries were captured by Dynabeads MyOne Streptavidin C1 magnetic beads (Invitrogen) and amplified. The resulting captured libraries were pooled in equal quantities and sequenced on one Illumina HiSeq X-10 lane in 150-bp paired-end mode.

### Orthologous gene group (OGG) construction

Raw sequence reads were quality filtered, adapters were trimmed using Trimmomatic v0.33^57^, and the reads were then sorted to DNA samples based on library indexes. The filtered reads of each sample were aligned to the 21,331 *Deinagkistrodon acutus* reference CDSs using the MEM algorithm of Burrows-Wheeler Aligner version 0.7.10^58^ with the default setting (-B 4) for viper species and relaxed setting (-B 2) for other snake species. The read mapping rate and the sequencing depth for each sample were calculated with SAMtools v1.4^59^. Based on the BWA mapping results, the consensus sequences for each sample were assembled and called using SAMtools (mpileup command with -q 5 -Q 10 -B -uf) and BCFtools v1.4 (call command with -c). Using this bioinformatic pipeline, we obtained the orthologous CDSs of each sample corresponding to the 21,331 *Deinagkistrodon acutus* reference CDSs. For each reference CDS of *Deinagkistrodon acutus*, the obtained orthologous CDSs from different samples had exactly the same length, and the coordinates of these CDSs matched exactly those of the reference sequence.

Because some snake genome data used in this study were not well annotated, we used a so-called “pseudo sequencing strategy” to identify orthologous CDSs from snake species with published genome data. Briefly, the raw genome sequences of each snake species were artificially fragmented into small ‘reads’ of 150 bp at 10 × tiling density. These “pseudo” reads were mapped to the *Deinagkistrodon acutus* reference CDSs and used to call orthologous CDSs using the aforementioned bioinformatic pipeline.

Finally, all the orthologous sequences for each reference CDS were put into a separate FASTA file as an orthologous gene group (OGG). The obtained OGG files were aligned using the ClustalW algorithm implemented in MEGA v6^60^ according to the translated protein sequences. The program PAL2NAL v14^61^ was used to convert the protein sequence alignments into corresponding codon alignments for each OGG.

### Phylogenetic analysis

To construct a genome-scale phylogeny for the 19 snake species sampled in this study, the protein alignments of all 21,331 OGGs were concatenated. The supermatrix was 11,231,292 amino acids in length with a data completeness of 90.3%. The phylogeny was reconstructed with maximum likelihood (ML) inference using RAxML v8.0^62^ under the GAMMA + WAGF model. Branch support was estimated by a rapid bootstrap algorithm (-f a option) with 500 replicates.

### Branch length analysis

ML trees were separately reconstructed for the 21,331 OGGs based on their protein alignments using RAxML v8.0 with the GAMMA + WAGF model. To make branch lengths comparable across different OGG trees, we enforced the genome tree (Fig. 1a) as a topology constraint during tree inference (-g option) and estimated branch lengths.

Our branch length analysis aimed to search for genes that evolved faster in pit vipers (Crotalinae) than in the comparative snake group, totally including three steps of comparison: the first step compares Crotalinae (pit vipers) and Colubridae + Elapidae (non IR-sensitive snakes); the second step compares Crotalinae (pit vipers) and Viperinae + Azemiopinae (non IR-sensitive viper snakes); and the third step compares the ancestor of pit vipers and extant Viperinae and Azemiopinae (Fig. 2).The average branch length calculation was performed using an in-house Python script and data plotting was performed using R. To avoid repetition, the logic of these comparisons and their purposes are not described here but are given in the corresponding Results section.

### Selection pressure analysis

Selection pressure analysis was performed using the CODEML program of the PAML package^39^. To reduce the impact of missing data, all the OGG codon alignments were first filtered to exclude species with more than 20% missing data. Only OGG codon alignments with more than three pit vipers and three non-pit snake species were used in the selection pressure analysis. For each OGG codon alignment, a separate user-tree was generated by pruning the corresponding missing species from the genome tree and labeling the ancestral branch of the pit viper lineage as the foreground branch. The two-ratio branch model (one ω was calculated for the foreground branch and another ω for all background branches; parameter setting "model = 2, NSsites = 0, fix_omega = 0") was compared to the one-ratio branch model (estimated only one ω for all branches; parameter setting "model = 0, NSsites = 0, fix_omega = 0"). After filtering out extremely low dN and dS values, the statistical *p*-values were calculated by likelihood ratio test (LRT) with one degree of freedom and false discovery rates (FDR) were computed using the Benjamini–Hochberg procedure^63^. This analysis searches for genes that experienced significant selection pressure alteration in the early evolution of pit vipers compared to other snakes.

For genes showing signs of selection pressure alteration, we used the branch-site model in PAML (parameter setting “model = 2, NSsites = 2”) to identify positively selected sites in the pit viper lineage. The null model (fix_omega = 1, omega = 1; all codons evolved neutrally) was compared with the alternative model (fix_omega = 0, omega = 1.5; some codons on the foreground branch experienced positive selection). The probable positively selected sites were identified and scored under Bayes empirical Bayes (BEB) probability.

### Gene ontology analysis

To provide an overview of the over-represented functional categories of identified genes, we performed GO enrichment analyses with DAVID 6.7 (https://david.abcc.ncifcrf.gov/) using lists of all candidate genes that we identified from both selection pressure analysis and branch length analysis. Enrichment was determined using the Fisher’s exact test and all P-values were adjusted for multiple comparisons by the Bonferroni method. Enriched GO categories were called using a 0.05 significance threshold and a minimum of 2 mapping entries.

### Gene expression analysis

We generated new RNA-seq data for two types of central nerve tissues (cerebrum and midbrain) for a pit viper *Gloydius brevicaudus* and another comparable no-pit snake *Lycodon rufozonatus*. Total RNA was extracted from 200 mg of fresh nervous tissues using the RNA prep Pure Tissue Kit (Tiangen, Beijing). mRNA isolation, library construction, sequencing, data cleaning and quality control were performed by BGI (Shenzhen, China). All libraries were sequenced on an Illumina HiSeq2000 sequencer. We obtained approximately 3.5 Gb of 90-bp paired-end read data (Q ≥ 20) for each tissue sample. RNA-seq data of peripheral nerve tissues (TG and DRG) were downloaded from a previous study^18^ (accession no. PRJNA121945; rattlesnake *Crotalus atrox* and combination data of rat snake *Elaphe obsoleta lindheimeri* and western coachwhip *Masticophis flagellum testaceus*. De novo transcriptome assembly was performed using Trinity v2014-07-17^64^ with default parameters. And then homologous sequences were identified through a bidirectional BLAST (e-value ≤ 10^−10^). Reads from individual tissue libraries were mapped to the corresponding transcriptome assembly by Bowtie2 v. 2.2.6^65^ with default parameters. Read counting for each transcript was performed by RSEM v. 1.3.0^66^. Raw read counts were then scaled and normalized per sample by transforming to FPKM values, and cross-sample normalization was performed using a trimmed mean of M values (TMM^67^) for comparison between libraries and species. Scaling and normalization were performed using the Trinity scripts “align_and_estimate_abundance.pl” and “abundance_estimates_to_matrix.pl”, respectively.

### Structural modeling

The structural models of two snake KCNK4 channels (pit viper *Deinagkistrodon acutus* and true viper *Vipera berus*) were generated based on the structure of human KCNK4 (PDB: 3UM7), with which these proteins share approximately 62% sequence identity. The SWISS-MODEL server (https://swissmodel.expasy.org/) was used to perform homology modeling of the dimer complex. The N terminus (amino acids 1–33) and C terminus (amino acids 299–464) were removed because no reliable coordinates are available for these regions in human KCNK4. The resulting 3D structures were graphically represented using PyMOL (https://www.pymol.org) and Chimera^68^.

## Supplementary information

Supplementary information.

Supplementary tables.

## Data Availability

The sequence capture reads generated in this study were deposited in the NCBI Short Read Archive under the BioProject accession number PRJNA511926, and the RNA-seq reads were deposited under the BioProject accession number PRJNA512390. All sequence alignments have been deposited as part of a Figshare project and are available at the following URL: https://figshare.com/s/3d20ad7188b9070e7a1f.
