# Supplementary material for: Whole-exome sequencing and genome-wide evolutionary analyses identify novel candidate genes associated with infrared perception in pit vipers
Source: Sci Rep. 2020 Aug 3;10:13033. doi: 10.1038/s41598-020-69843-w (PMC7400743; doi:10.1038/s41598-020-69843-w)
Supplement: Supplementary file 1 — Supplementary information. [file 41598_2020_69843_MOESM1_ESM.pdf]

Title：Whole-exome sequencing and genome-wide evolutionary analyses identify novel candidate genes associated with infrared perception in pit vipers

Authors: Na Tu, Dan Liang, Peng Zhang

List of supplementary files:

Supplementary Figure S1. Individual gene trees of the 38 candidate genes identified by branch length analysis (colored in red), and a genome-scale tree used as the negetive control (colored in blue). Trees were reconstructed using the Maximum likelihood (ML) methods on protein sequences. All trees were drawn to same scale, with branch length measured to the proportion of substitutions per site. R_1, R_2 and R_3 represent the ratio of the branch length of pit vipers to that of other groups from the three screening steps. The ancestral branches of pit vipers are indicated in bold lines.

Supplementary Figure S2. Protein alignment of *KCNK4* gene of 19 snakes and positively selected sites (PSSs) identified by PAML. PSSs were detected by CodeML under the Branch-site model and scored under Bayes empirical Bayes. The common ancestral branch leading to all pit vipers was set as the foreground branch. These sites are numbered according to the full *Deinagkistrodon acutus* KCNK4 protein sequence and indicated by red boxes. Amino acid residues that are highly conserved in all other snakes, but evolved to a different state in pit vipers are specially marked with red triangles. Missing amino acid sites are indicated as X.

Supplementary Table S1. Snake sample information and whole-exome capture statistics.

Supplementary Table S2. Genome data sources used in this study.

Supplementary Table S3. Candidate genes identified by the branch length analysis.

Supplementary Table S4. Selection pressure analysis results estimated with PAML using branch-specific model.

Supplementary Table S5. Genes with significantly elevated omega values in the ancestral branch of pit viper lineage.

Supplementary Table S6. Number of pit viper-specific amino acid substitutions of the 47 candidate genes.

Supplementary Table S7. The expression levels of candidate genes in four nerve tissues of both pit vipers and nonpit snakes.

Supplementary Figure S1. Individual gene trees of the 38 candidate genes identified by branch length analysis (colored in red), and a genome-scale tree used as the negetive control (colored in blue). Trees were reconstructed using the Maximum likelihood (ML) methods on protein sequences. All trees were drawn to same scale, with branch length measured to the proportion of substitutions per site. R_1, R_2 and R_3 represent the ratio of the branch length of pit vipers to that of other groups from the three screening steps. The ancestral branches of pit vipers are indicated in bold lines.

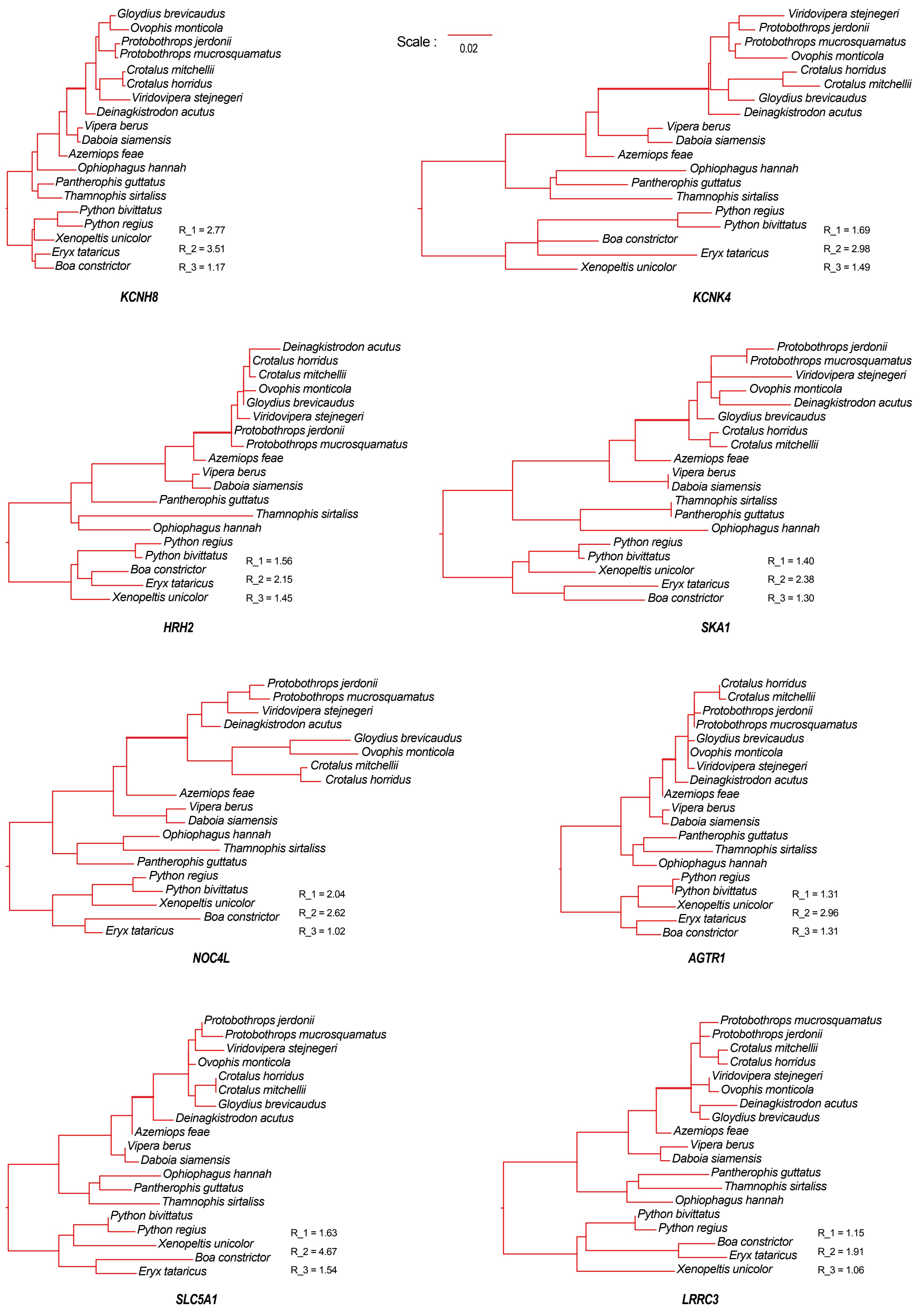

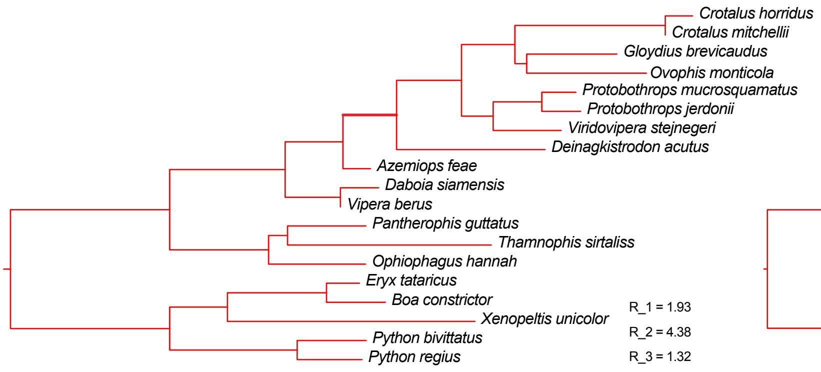

F1NPK8

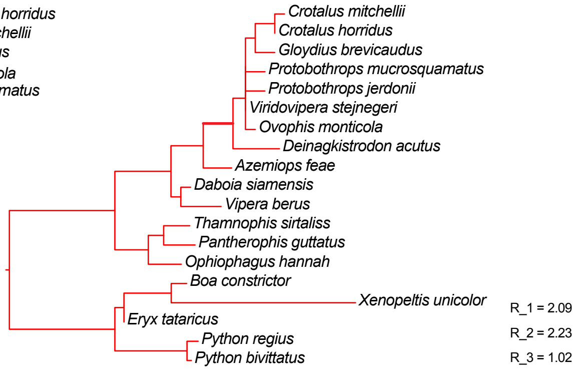

COQ5

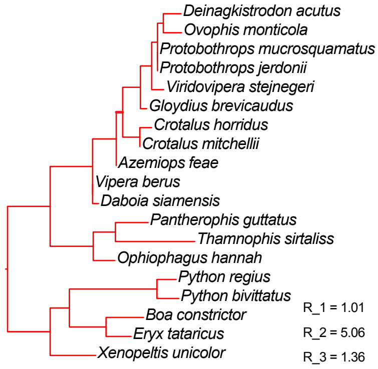

MYOT

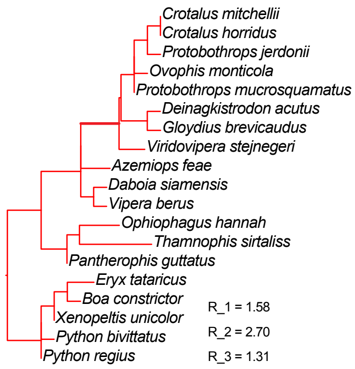

FAM58A

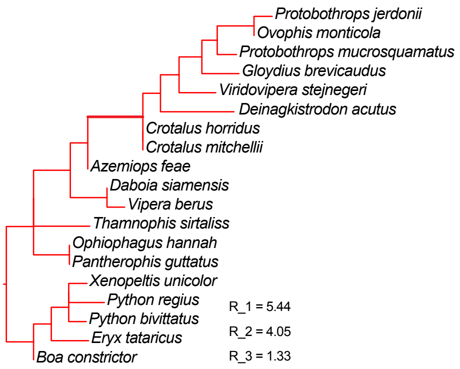

CHMP6

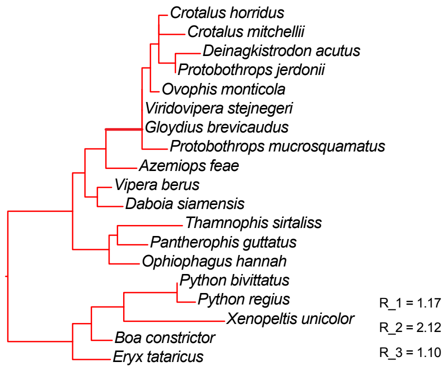

HABP4

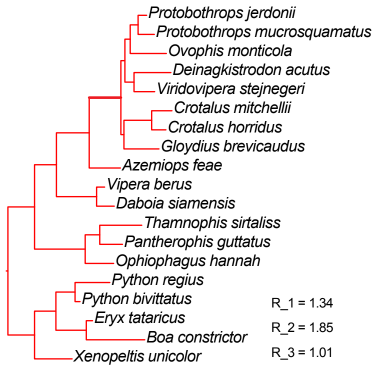

WAPAL

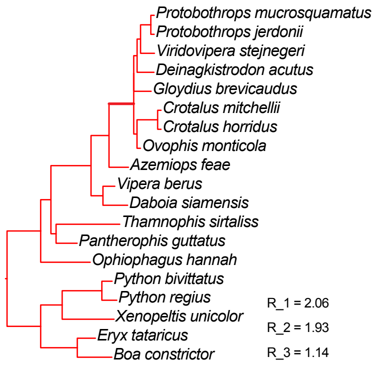

ADAMTS2

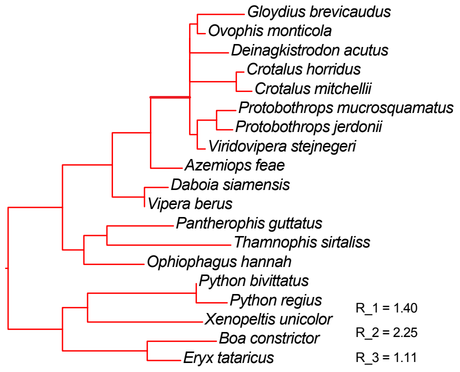

GK5

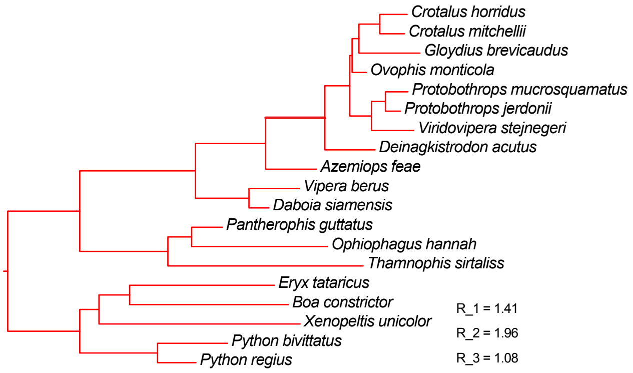

CAST

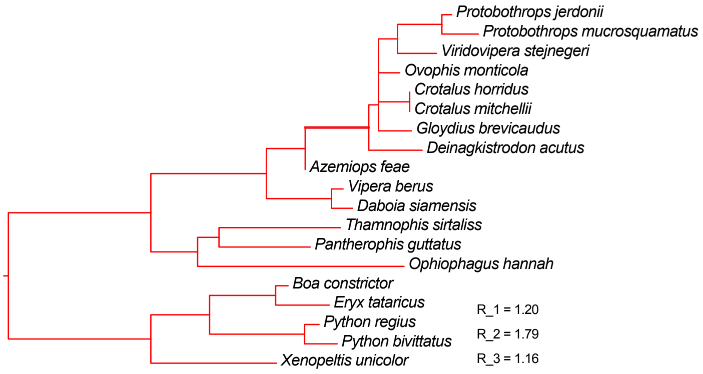

METTL20

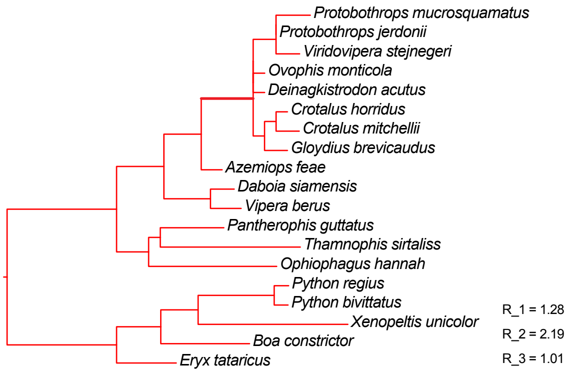

KCNK17

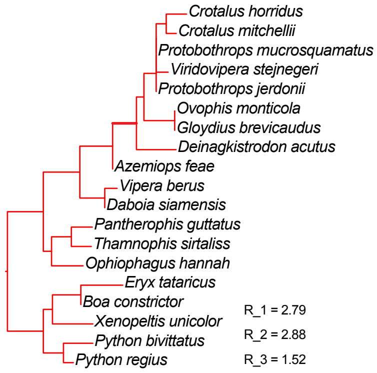

C1QL2

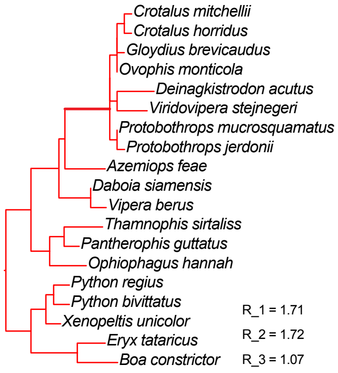

ZNT6

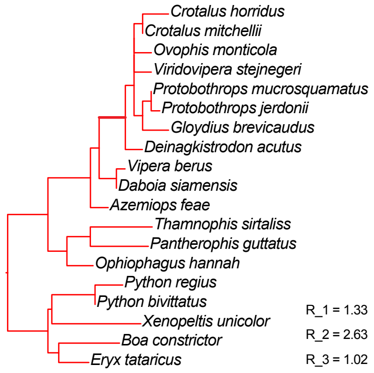

SETD7

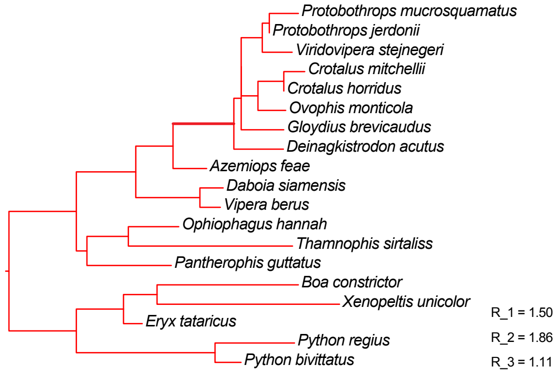

CYBB

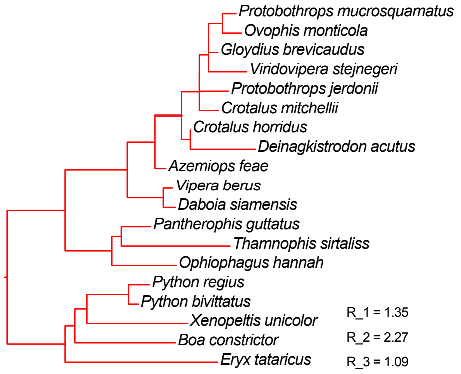

ZGC_136493

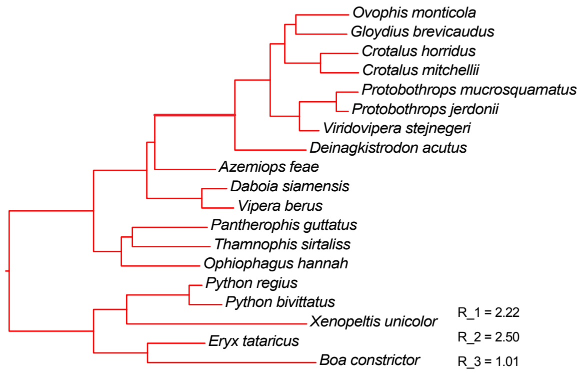

Q5ZLE7

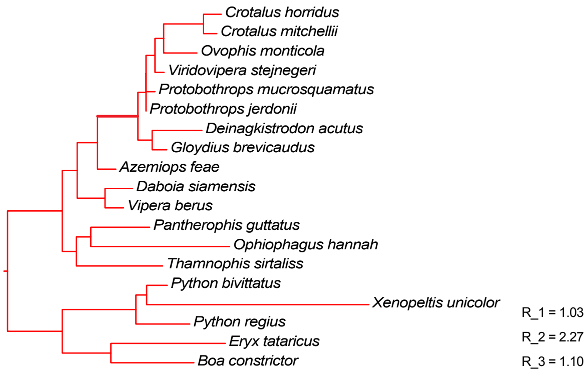

WWOX

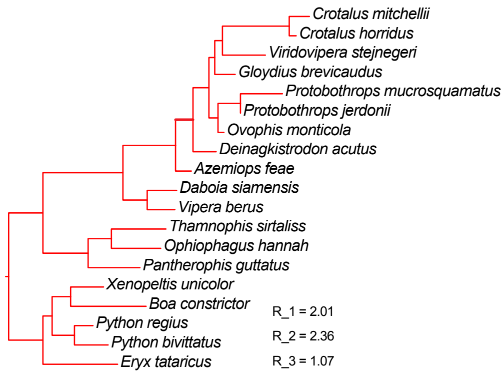

ZNF703

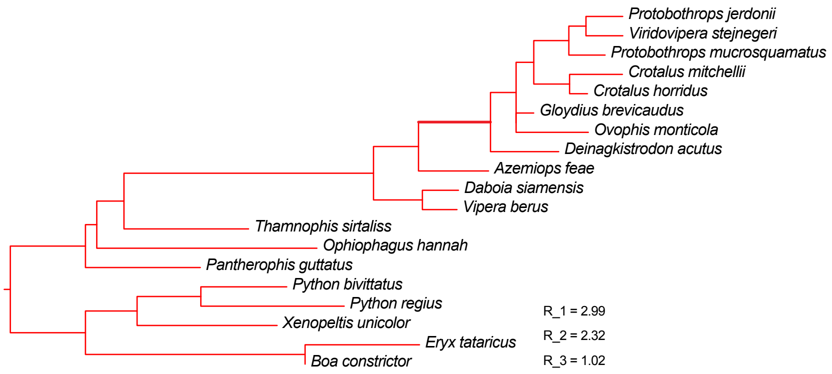

CRYAB

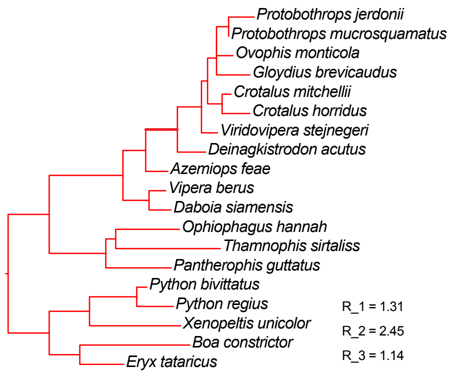

SLC45A1

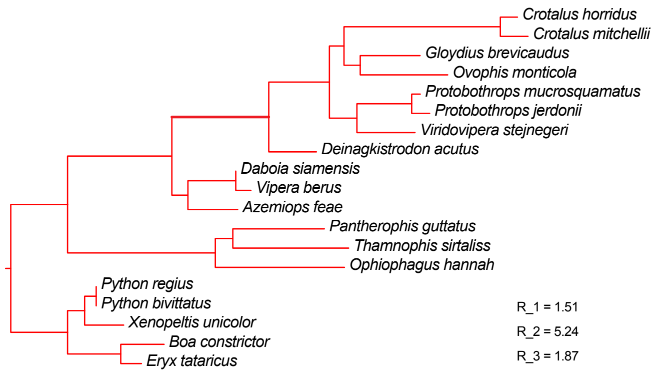

XB-GENE-5885309

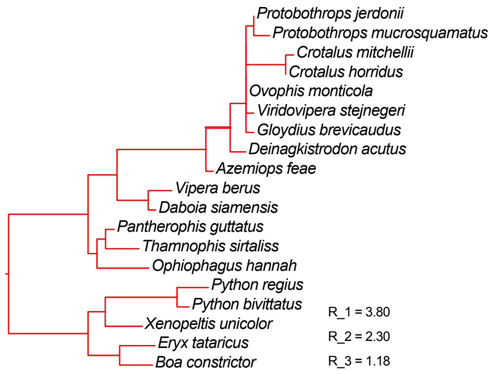

CERS6

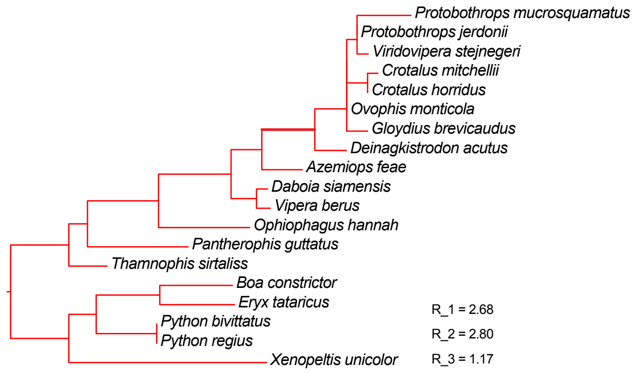

DECR2

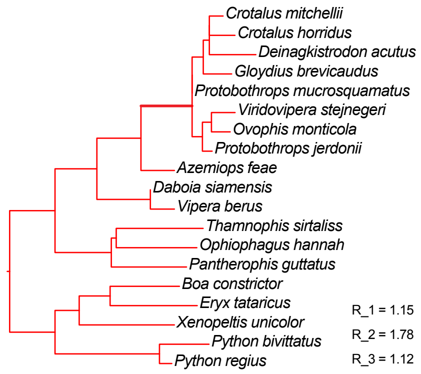

SLC6A16

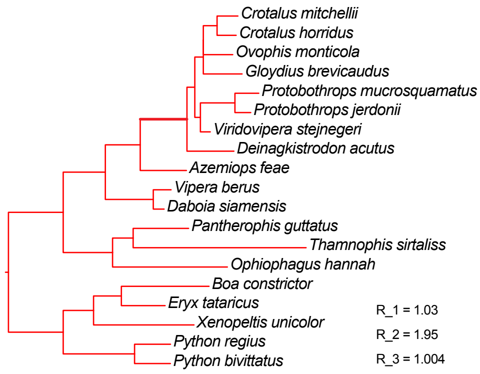

VWA7

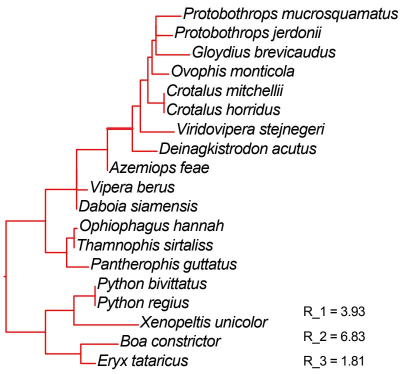

LHX3

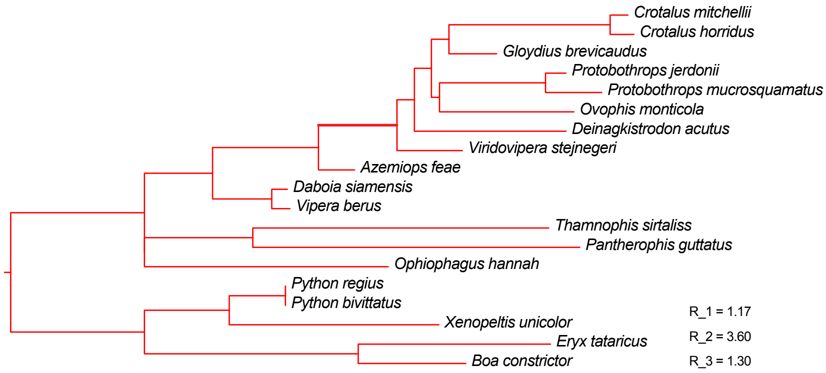

NDUFB8

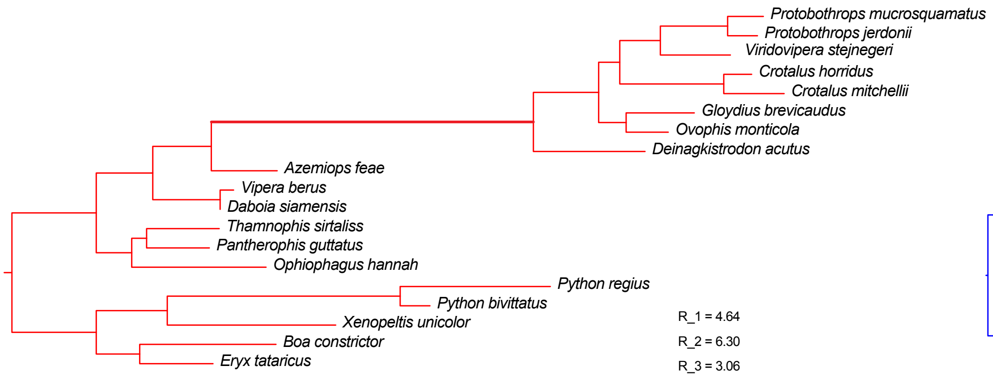

TRPA1

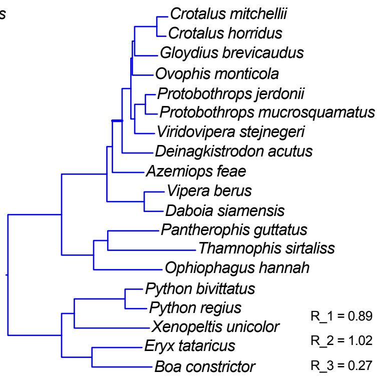

Genome-scale tree

Supplementary Figure S2. Protein alignment of *KCNK4* gene of 19 snakes and positively selected sites (PSSs) identified by PAML. PSSs were detected by CodeML under the Branch-site model and scored under Bayes empirical Bayes. The common ancestral branch leading to all pit vipers was set as the foreground branch. These sites are numbered according to the full *Deinagkistrodon acutus* KCNK4 protein sequence and indicated by red boxes. Amino acid residues that are highly conserved in all other snakes, but evolved to a different state in pit vipers are specially marked with red triangles. Missing amino acid sites are indicated as X.

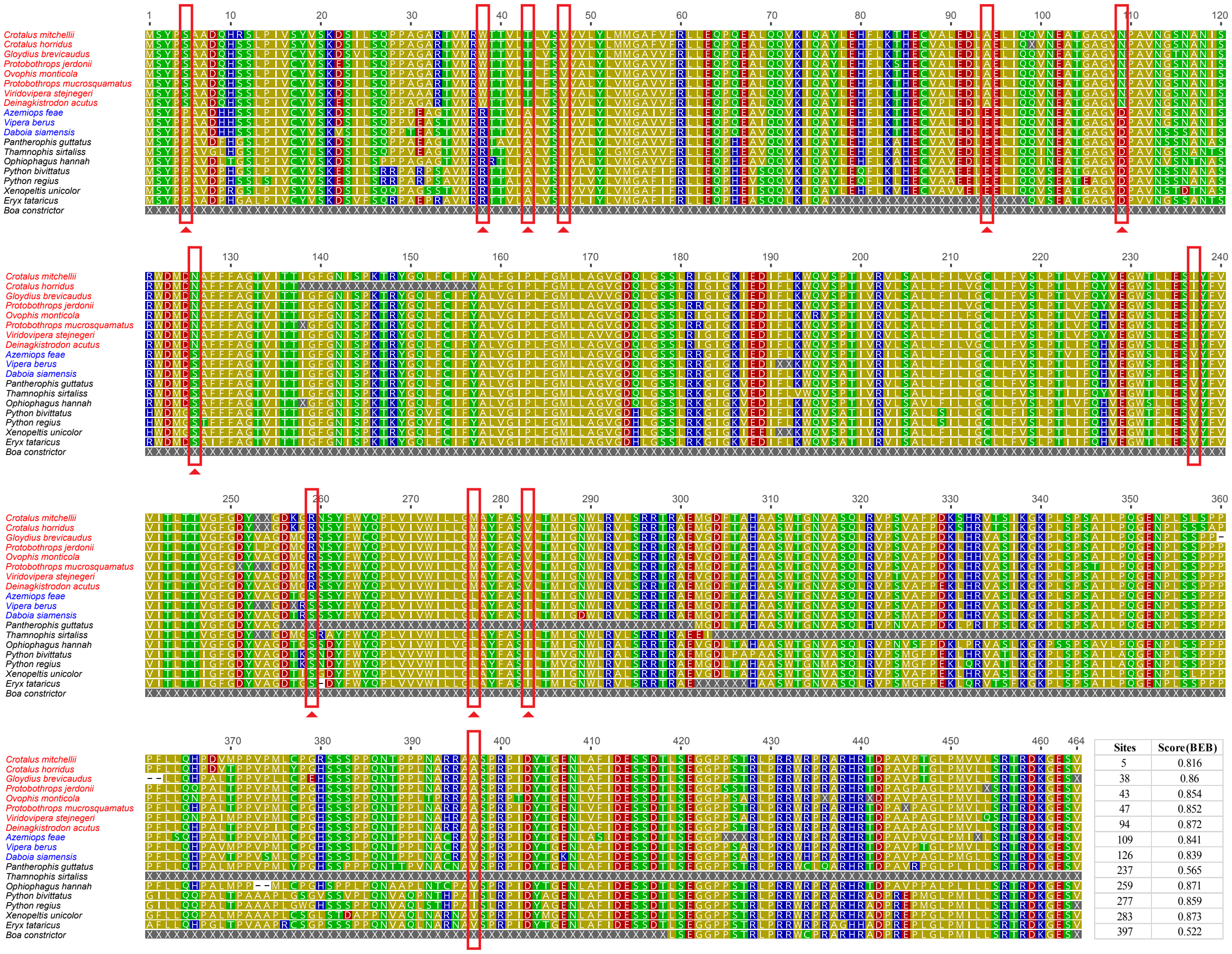

Sites	Score(BEB)
5	0.816
38	0.86
43	0.854
47	0.852
94	0.872
109	0.841
126	0.839
237	0.565
259	0.871
277	0.859
283	0.873
397	0.522

Table S1. Snake sample information and whole-exome capture statistics

Species	Taxonomy	Collection locality	Pit-bearing	No. of read pairs	% Duplicated	% On Target	CDS retrieved	CDS completeness	Sequencing depth
Gloydius brevicaudus	Viperidae:Crotalinae	Shaoguan, Guangdong, China	yes	39,835,026	13.31	27.77	21,304	96.55%	69×
Protobothrops jerdonii	Viperidae:Crotalinae	Tianquan, Sichuan, China	yes	31,289,144	11.64	28.78	21,294	96.43%	57×
Viridovipera stejnegeri	Viperidae:Crotalinae	Yongzhou, Hunan, China	yes	40,128,419	13.40	31.25	21,300	97.53%	72×
Ovophis monticola	Viperidae:Crotalinae	Mengzhi, Yunnan, China	yes	43,254,034	11.92	30.43	21,314	97.74%	77×
Azemiops feae	Viperidae:Azemiopinae	Conghua, Guangdong, China	no	26,719,194	13.11	28.48	21,207	93.57%	52×
Daboia siamensis	Viperidae:Viperinae	Shaoguan, Guangdong, China	no	49,481,425	11.23	28.13	21,213	94.23%	70×
Ophiophagus hannah	Elapidae	Shaoguan, Guangdong, China	no	33,316,966	11.79	20.7	21,187	90.20%	39×
Python regius	Pythonidae	Private breeding	yes	30,086,726	11.21	9.8	20,865	82.61%	17×
Xenopeltis unicolor	Xenopeltidae	Mangshan, Hainan, China	no	55,966,938	10.00	12.43	21,167	87.37%	35×
Eryx tataricus	Boidae	Private breeding	no	52,431,658	10.15	11.8	21,117	86.15%	27×

Table S2. Genome data sources used in this study (newly generated data are in blue, published genomes are in black).

Species	Taxonomy	Pit-bearing	Data type	Data deposition	Accession Numbers or Released Version	No.of CDS retrieved	CDS completeness%
Crotalus mitchellii	Viperidae:Crotalinae	yes	Genome	NCBI SRA	GCA_000737285.1 (CrotMitch1.0)	21,154	94.98
Crotalus horridus	Viperidae:Crotalinae	yes	Genome	NCBI SRA	GCA_001625485.1 (ASM162548v1)	21,211	95.53
Deinagkistrodon acutus	Viperidae:Crotalinae	yes	Genome	http://dx.doi.org/10.5524/100196.	PRJNA314559	21,331	100
Gloydius brevicaudus	Viperidae:Crotalinae	yes	Sequence capture	NCBI SRA	SAMN10651530	21,304	96.55
Ovophis monticola	Viperidae:Crotalinae	yes	Sequence capture	NCBI SRA	SAMN10651533	21,314	97.74
Protobothrops jerdonii	Viperidae:Crotalinae	yes	Sequence capture	NCBI SRA	SAMN10651531	21,294	96.43
Protobothrops mucrosquam	Viperidae:Crotalinae	yes	Genome	NCBI SRA	GCA_001527695.3 (P.Mucros_1.0)	21,271	97.79
Viridovipera stejnegeri	Viperidae:Crotalinae	yes	Sequence capture	NCBI SRA	SAMN10651532	21,300	97.53
Azemiops feae	Viperidae:Azemiopinae	no	Sequence capture	NCBI SRA	SAMN10651534	21,207	93.57
Daboia siamensis	Viperidae:Viperinae	no	Sequence capture	NCBI SRA	SAMN10651535	21,213	94.23
Vipera berus	Viperidae:Viperinae	no	Genome	NCBI SRA	GCA_000800605.1 (Vber.be_1.0)	20,620	91.46
Pantherophis guttatus	Colubridae	no	Genome	NCBI SRA	GCA_001185365.1 (PanGut1.0)	20,715	88.69
Thamnophis sirtaliss	Colubridae	no	Genome	NCBI SRA	GCA_001077635.2 (Thamnophis_sirtalis-6.0)	20,164	77.26

Ophiophagus hannah	Elapidae	no	Sequence capture	NCBI SRA	SAMN10651536	21,187	90.2
Python bivittatus	Pythonidae	yes	Genome	NCBI SRA	GCA_000800605.1 (Python_molurus_bivittatus-5.0.2)	19,515	81.05
Python regius	Pythonidae	yes	Sequence capture	NCBI SRA	SAMN10651537	20,865	82.61
Xenopeltis unicolor	Xenopeltidae	no	Sequence capture	NCBI SRA	SAMN10651538	21,167	87.37
Boa constrictor	Boidae	no	Genome	platanus.bio.titech.ac.jp/platanus-assembler/ platanus-1-2-1	---	19,557	80.85
Eryx tataricus	Boidae	no	Sequence capture	NCBI SRA	SAMN10651539	21,117	86.15
